# Renal Alterations in Feline Immunodeficiency Virus (FIV)-Infected Cats: A Natural Model of Lentivirus-Induced Renal Disease Changes

**DOI:** 10.3390/v4091372

**Published:** 2012-08-27

**Authors:** Alessandro Poli, Natasa Tozon, Grazia Guidi, Mauro Pistello

**Affiliations:** 1 Department of Animal Pathology, Prophylaxis and Food Hygiene, Veterinary Faculty, University of Pisa, Viale delle Piagge 2, Pisa 56124, Italy; Email: apoli@vet.unipi.it; 2 Clinic for Small Animal Medicine and Surgery, Veterinary Faculty, University of Ljubljana, Gerbičeva 60, Ljubljana 1000, Slovenia; Email: Natasa.Tozon@vf.uni-lj.si; 3 Department of Veterinary Clinic, Veterinary Faculty, University of Pisa, Via Livornese, San Piero a Grado, Pisa 56122, Italy; Email: guidi@vet.unipi.it; 4 Department of Experimental Pathology, University of Pisa, Via S. Zeno, 35/39, Pisa 56127, Italy

**Keywords:** cat, feline immunodeficiency virus, FIV, kidney diseases, renal pathology

## Abstract

Human immunodeficiency virus (HIV) is associated with several renal syndromes including acute and chronic renal failures, but the underlying pathogenic mechanisms are unclear. HIV and feline immunodeficiency virus (FIV) share numerous biological and pathological features, including renal alterations. We investigated and compared the morphological changes of renal tissue of 51 experimentally and 21 naturally infected cats. Compared to the latter, the experimentally infected cats exhibited some mesangial widening and glomerulonephritis, milder proteinuria, and lower tubular and interstitial alterations. The numbers of giant protein tubular casts and tubular microcysts were also lower. In contrast, diffuse interstitial infiltrates and glomerular and interstitial amyloidosis were detected only in naturally infected cats. Similar alterations are found in HIV infected patients, thus supporting the idea of a causative role of FIV infection in renal disease, and underlining the relevance of the FIV and its natural host as an animal model for investigating lentivirus-associated nephropathy.

## 1. Introduction

Nephropathies frequently complicate the course of infection due to human immunodeficiency virus (HIV), most of them resulting from an underlying chronic renal disease [1]. The pathogenetic mechanisms involved in these disorders are still obscure: genetic factors and the renal cellular response, triggered by HIV proteins and an immune-activated microenvironment, are thought to play an important role. Transgenic animals and natural infectious models of HIV that recapitulate the human diseases are important tools for gaining insights into disease pathogenesis, genetic susceptibility and therapeutic intervention [2]. Chief among the natural models are simian immunodeficiency virus (SIV) and feline immunodeficiency virus (FIV), along with their respective hosts. Both SIV and FIV are lentiviruses closely related to HIV. They cause a chronic infection that mimics, to a large extent, HIV infection, including nephropathy. 

Along the course of infection, FIV induces immunological abnormalities characterized by a profound decline in the absolute number of the CD4^+^ T-cells, a consequent inversion of the CD4^+^/CD8^+^ T-cell ratio, and, possibly, an increased susceptibility to opportunistic infections, and various clinic-pathological conditions [3]. Like HIV, FIV is endemic and genetically heterogeneous. The circulating strains have been grouped into five subtypes, from A to E, which are unevenly distributed geographically and have an inter-subtype diversity >20% at an amino acid level [4]. Once transmitted by biting or, to a less extent, vertically or sexually, FIV establishes a life-long persistent infection in the face of strong humoral and cell-mediated immune responses that appear shortly after the initial viremic phase [4]. The acute phase of infection lasts a few days to a few weeks, and is asymptomatic in a large proportion of cats. Viral replication and clinical symptoms, if present, eventually subside and the animal enters in an asymptomatic period that typically lasts 4 to 6 years or remains clinically silent throughout animal’s life. In 30% of cats, and mostly influenced by cofactors and life-style [4], the infection proceeds to the last stage, the feline AIDS (F-AIDS), characterized by profound immunodeficiency, secondary infections sustained by various opportunistic pathogens, and host diseases. 

Clinical manifestation and disease outcome depend on a combination of concomitant or subsequent infections, which may interact with FIV or exacerbate its pathogenic potential. Some, mostly undefined, host genetic factors, and specific immune responses also contribute to clinical onset. Immunodeficiency combined with either local or systemic immunostimulation most frequently results in the emergence of severe forms of gingivostomatitis, chronic rhinitis, lymphadenopathy, weight loss and renal diseases [4].

Despite increasing knowledge of most of the clinical and pathological features, as far as we are aware there is no detailed description of the renal lesions found in naturally and experimentally FIV-infected cats. The aim of our study was thus to investigate the histological renal alterations in 51 cats experimentally inoculated with subtype A FIV-Pet and subtype B FIV-M2 strains, and sacrificed at different times post-infection (pi). Findings were compared to those observed in 21 naturally FIV-infected cats. Given the similarities with HIV-infected patients, we believe that the feline animal model is an excellent means for advancing knowledge of HIV-associated renal diseases.

## 2. Results and Discussion

Characteristic and clinical conditions of study animas are shown in Table 1. The 21 naturally FIV-infected cats were older compared to experimentally infected subjects and controls (median ages statistically difference at *p* < 0.01). Fifteen of the naturally infected cats were males and 6 females. Of these, three females and one male were neutered, remaining 17 were intact. Finally, ten of these were in the asymptomatic phase (clinical staging 3), while the other eleven were symptomatic (clinical staging 4). Experimentally FIV-infected cats and controls were all intact females and aged between 2 and 6 years at time of analysis. At time of sacrifice, these subjects were all healthy except one infected with FIV-M2 strain.

**Table 1:** Characteristics and creatinine and urine protein concentrations of study cats.

Characteristic	Naturally Infected (n = 21)	Experimentally Infected			Controls (n = 4)
		FIV-Pet (n = 17)	FIV-M2 (n = 28)	FIV-Pet + FIV-M2 (n = 6)	
	Median (Range)	Median (Range)	Median (Range)	Median (Range)	Median (Range)
Age (years)	8.0 (4.0–13.0)	3.0 (2.0–6.0)	4.0 (2.0–6.0)	4.5 (4.0–5.0)	4.5 (4.0–6.0)
Sex					
Male	15				
Female	6	17	28	6	
Neuter status					
Intact	17	17	28	6	
Altered	4				
Clinical status					
Sick	11		1		
Healthy	10	17	27	6	
Creatinine concentration	120 (73–709)	123 (100–131)	118 (93–144)	121 (103–139)	107 (95–125)
UPC	1.17 (0,18–14.00)	0.69 (0.31–7.00)	0.52 (0.25–18.00)	0.92 (0.55–13.00)	0.25 (0.17–0.31)

**Figure 1:**
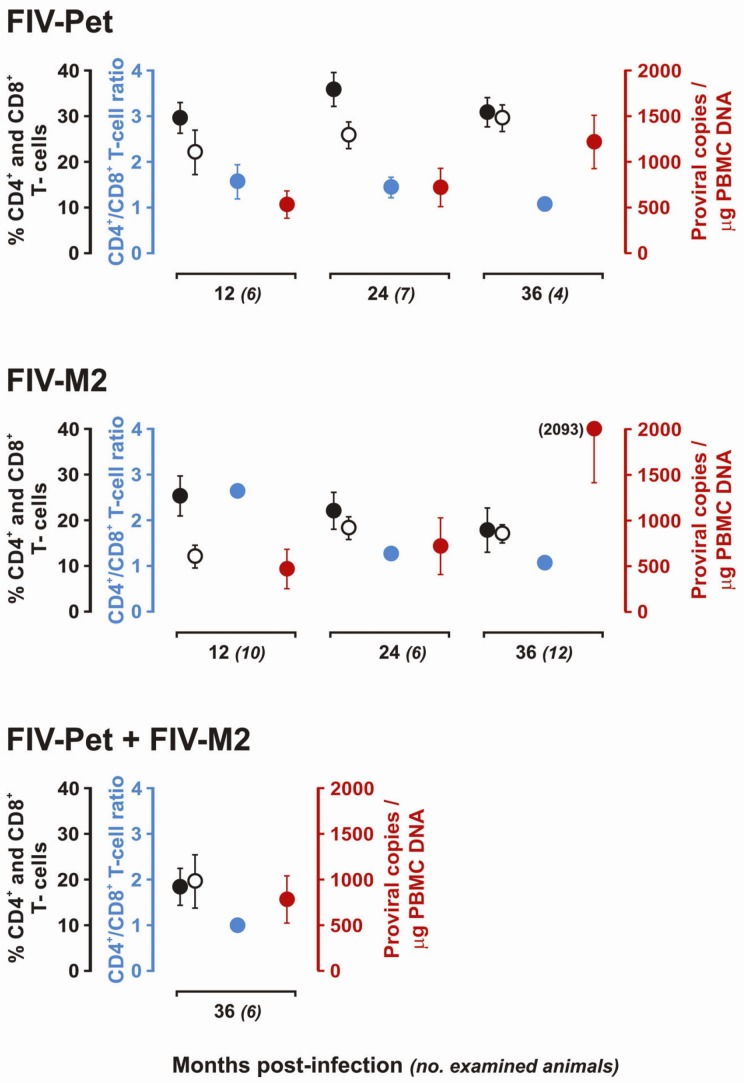
Analysis of CD4^+^ and CD8^+^ T-cell levels and proviral load in the peripheral blood of the examined cats at different times from infection. Solid and empty black circles indicate percent of CD4^+^ and CD8^+^ T-cells, respectively. Light blue circles indicate the CD4^+^ and CD8^+^ T-cell ratio that was calculated by dividing the CD4^+^ value by the CD8^+^ value. Red circles indicate the proviral load expressed as numbers of proviruses per µg of PBMC DNA and determined by competitive or real-time PCRs. The proviral load values for FIV-Pet + FIV-M2 cats are the total number of FIV-Pet and FIV-M2 proviral genomes. Whiskers indicate the standard deviation.

Figure 1 shows the CD4^+^/CD8^+^ T-cell counts and viral load at the time of sacrifice in the experimentally infected subjects. In these animals a progressive decline of CD4^+^ T-cell and a reduced CD4^+^/CD8^+^ T-cell ratio were observed. This was particularly evident in the subjects sacrificed ≥36 months pi. In contrast, the proviral load in peripheral blood mononuclear cells (PBMCs) constantly increased over time. Although competitive and real-time PCR assays to quantitate the FIV RNA genome yielded different and difficult to compare results, increased levels of plasma viremia relative to time of infection were also observed within FIV-Pet and FIV-M2 groups (data not shown). These results indicated that, notwithstanding the absence of overt clinical symptoms, the FIV infection progressed and eroded immune system efficiency in all infected animals irrespectively of the infecting strains. In fact, no significant differences in lymphocyte subset changes and proviral load were observed between FIV-Pet and FIV-M2 groups. Interestingly, FIV-Pet + FIV-M2 group had CD4^+^ and CD8^+^ T-cell levels and ratios comparable to animals infected three years earlier with either two viruses alone but the total proviral load in PBMCs, *i.e*., FIV-Pet plus FIV-M2 genomes first determined by competitive PCR then reexamined by real-time PCR, was lower compared to FIV-Pet and FIV-M2 groups. 

As described in a previous paper [5], these animals were preinfected with scaled doses of a FIV-Pet that had been cultivated *in vitro* and had lost most of its pathogenic potential, and which thus established a low-grade infection in all the inoculated animals. Seven months later animals were challenged with a fully virulent strain of FIV-M2 and monitored for over three years. The results revealed that preinfection with subtype A FIV-Pet did not prevent superinfection and nor did the acute phase of infection give rise to subtype B FIV-M2. However, two years post FIV-M2 inoculation, FIV-Pet preinfection significantly prevented the increase in viral burden compared to control cats infected in parallel with FIV-M2 [5]. The reduced viral burden observed one year later, when the animals were sacrificed to analyze viral distribution and histopathology in tissues, are thus in line with our follow-up results.

Histopathological examinations of renal tissues showed glomerular changes in 18/21 (85.7%) of the naturally and in 26/51 (51.0%) of the experimentally FIV-infected cats. No alterations were detected in controls (Table 2).

**Figure 2:**
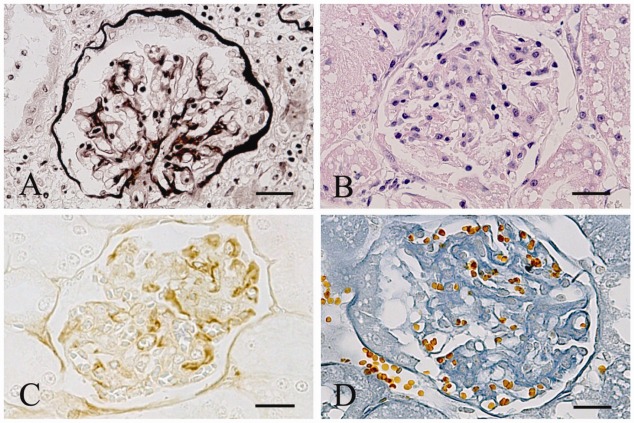
Representative results of renal lesions observed in FIV-M2-infected cats. (**A**) Mesangial widening. Mild increase in mesangial matrix with a minimal increase in intraglomerula cellularity. Jones’ periodic acid-silver methenamine stain. (Bar = 80 µm); (**B**) Segmental mesangioproliferative glomerulonephritis. Scattered areas of mild proliferation of mesangial cells with scanty inflammatory infiltrates. Hematoxylin-Eosin stain. (Bar = 80 µm); (**C**) Mesangioproliferative glomerulonephritis. Segmental deposition of IgG. Strepavidin biotin peroxidise complex method, Mayer’s hematoxylin counterstain. (Bar = 80 µm); (**D**) Membranoproliferative glomerulonephritis. Mesangial enlargement and thickening of capillary walls with tram track appearance. Azan trichromic stain. (Bar = 80 µm).

Mesangial widening, characterized by an increase in mesangial matrix and with or without segmental glomerulosclerosis (Figure 2A), was observed in 6/17 FIV-Pet infected cats (two 12 months pi, one 24 months pi, and three 36 months pi). One cat infected by 12 months and two cats infected by 24 months with FIV-Pet also showed focal and segmental mesangioproliferative glomerulonephritis (GN) (Figure 2B). In these areas, IgG (Figure 2C) and C3 deposits were detected by immunohistochemistry (IHC). In the cats infected with FIV-M2, the renal changes were detected in 12/28 subjects: 6 of these had mesangial widening (two infected by 12 months, one by 24 months, and three by ≥36 months), five were affected by focal and segmental mesangioproliferative GN (one cat each infected by 12 and 24 months, and 3 by ≥36 months). Finally, one cat infected by ≥36 months showed membranoproliferative GN. In this case, the mesangial cellularity had increased and presented enlarged and thickened capillary walls that caused “splitting” of glomerular basement membrane (Figure 2D). Five of the six cats infected with both strains and sacrificed at ≥36 months pi showed mesangial widening (two cats) and focal and segmental mesangioproliferative GN (three cats).

Six FIV-Pet, eleven FIV-M2, and three FIV-Pet + FIV-M2 infected cats showed degenerative changes in the tubular epithelial cells. Tubular microcysts as well as giant protein casts were occasionally observed. In particular, the tubular mycocyst were detected in four cats, two infected with FIV-M2 and in two with both viruses and giant protein casts in two cats infected with both viruses and sacrificed 36 months pi. Interstitial changes were also infrequent. Scattered periglomerular infiltrates were detected in one FIV-Pet cat sacrificed at 24 months pi, three infected with FIV-M2 (one sacrificed at 24 months and two sacrificed ≥36 months pi) and two cats infected with both viruses.

**Table 2:** Renal alterations detected in experimentally feline immunodeficiency virus (FIV)-infected cats sacrificed at the indicated times post-infection (pi).

Renal Alterations	Naturally Infected n = 21 (%)	Controls	12 Months pi		24 Months pi		≥36 Months pi		
			FIV-Pet n = 6 (%)	FIV-M2 n = 10 (%)	FIV-Pet n = 7 (%)	FIV-M2 n = 6 (%)	FIV-Pet n = 4 (%)	FIV-M2 n = 12 (%)	FIV-Pet + FIV-M2 n = 6 (%)
Mesangial widening	9 (42.9)	0	2 (33.3)	2 (20.0)	1 (14.3)	1 (16.6)	3 (75.0)	3 (25.0)	2 (33.3)
Glomerulo-nephritis	3 (14.3)	0	1 (16.6)	1 (10.0)	2 (28.6)	1 (16.6)	0	4 (33.3)	3 (50.0)
Glomerular amyloidosis	8 (30.1)	0	0	0	0	0	0	0	0
Tubular changes	10 (47.6)	0	2 (33.3)	2 (20.0)	1 (14.3)	2 (33.3)	3 (75.0)	7 (58.3)	3 (50.0)
Interstitial lesions	17 (81–0)	0	0	0	1 (14.3)	1 (16.6)	0	2 (16.7)	2 (33.3)
Interstitial amyloidosis	7 (33.3)	0	0	0	0	0	0	0	0
No alterations	3 (14.3)	4 (100)	3 (50.0)	7 (70.0)	4 (57.1)	4 (66.6)	1 (25.0)	5 (41.6)	1 (16.6)

**Figure 3:**
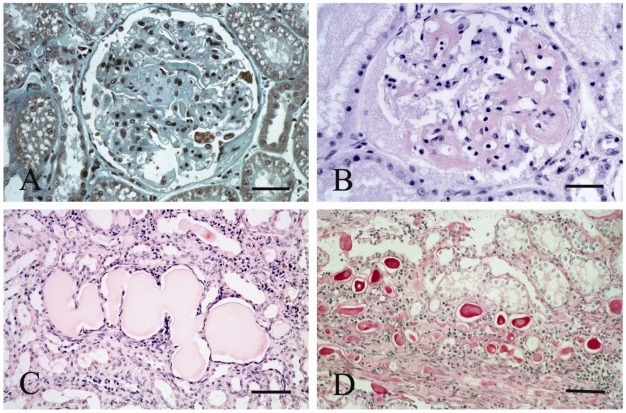
Representative results of renal lesions observed in naturally FIV-infected cats. (**A**) Mesangial widening with segmental glomerulosclerosis. Increase in mesangial matrix with a minimal increase in intraglomerular cellularity. Protein droplets were detectable within podocyte cytoplasmas. Azan trichrome stain. (Bar = 80 µm); (**B**) Glomerular amyloidosis. Diffuse increase of capillary walls due to amyloidosis depostion. Congo red stain. (Bar = 80 µm); (**C**) Tubular mycrocysts. Presence of interstitial infiltration and dilated tubules forming tubular mycrocysts. Hematoxylin-Eosin stain. (Bar = 80 µm); (**D**) Giant protein cats. PAS-positive proteinacous cats within tubular microcysts. PAS stain. (Bar = 80 µm).

Glomerular changes were detected in 18/21 naturally infected subjects. Mesangial matrix with occasional segmental glomerulosclerosis was observed in 9/21 cats (Figure 3A), in this case protein droplets were frequently detected within podocytes. Immune-mediated GN of mesangioproliferative type in 3/21 cats, and amyloid deposition was present in 8/21 cats (Figure 3B). Amyloid deposits were segmental and focal in six cases and diffuse in two. In all cases the amyloid deposits were KMnO4 sensitive. Tubulointerstitial alterations were also a common finding in the naturally infected cats: degeneration of tubular epithelial cells was observed in ten cats, tubular microcysts in eight (Figure 3C), and giant protein tubular casts (Figure 3D) in four subjects. Interstitial alterations were also frequent and consisted of interstitial infiltration by lymphocytes and plasma cells. Infiltration was scanty periglomerular (eight subjects), diffuse without fibrosis (six), and diffuse with interstitial fibrosis (three). Interstitial amyloidosis was detected in seven subjects, while no interstitial alterations were detected in four cats. Like above, the amyloid deposit was KMnO4 sensitive. Table 3 summarizes the results of IHC analyses in experimentally and naturally infected cats.

**Table 3:** Main immunohistochemical findings in naturally and experimentally FIV-infected cats and controls.

Immunohistochemistry	Control Cats n = 4 (%)	Naturally Infected cats n = 21 (%)	Experimentally Infected Cats		
			FIV-Pet n = 17 (%)	FIV M2 n = 28 (%)	FIV-Pet + FIV M2 n = 6 (%)
IgG deposits in mesangium	0	3 (14.3)	3 (17.6)	5 (17.9)	3 (50.0)
IgG deposits in capillary loops	0	1 (04.8)	1 (05.9)	2 (07.1)	2 (33.3)
IgM deposits	0	14 (66.7)	6 (35.3)	6 (21.3)	3 (50.0)
IgA deposits	0	0	0	0	0
C3 deposits	0	14 (66.7)	6 (35.3)	6 (21.3)	3 (50.0)
Mouse monoclonal anti-human AA amyloid	0	8 (38.1)	0	0	0
Rabbit polyclonal anti-feline AA amyloid	0	8 (38.1)	0	0	0
Rabbit polyclonal anti-feline AL amyloid	0	0	0	0	0

No immunoglobulins or C3 deposits were detected in the uninfected control cats. Both in experimentally and naturally FIV-infected cats, glomeruli affected by GN showed segmental and predominantly mesangial granular deposits of IgG, IgM and C3, while rarely scattered deposits were detected along the capillary loops, IgA staining was not observed. Large proteinaceous casts were positive for IgG and weak for IgA. Amyloid deposits were always positive for the mouse monoclonal anti-human AA and the rabbit polyclonal against the feline AA amyloid, while they were always negative for the rabbit polyclonal anti-AL amyloid.

Mesangial widening, GNs and tubular alterations were detected both in experimentally and naturally infected cats. Giant protein tubular casts and tubular microcysts were more frequently detected in naturally than experimentally infected subjects (*p* < 0.05). Interstitial alterations were also more frequent in naturally compared to experimentally infected cats (*p* < 0.001). Further, the former group presented glomerular and interstitial amyloid deposits that were not detected in the experimentally infected ones (*p* < 0.001). It should be mentioned, however, that a few naturally FIV-infected subjects were old and part of these renal changes, in particular the interstitial ones, could be aged related.

Similarly to previous studies [6,7], these results demonstrate that the experimentally FIV infected cats had renal changes similar, to some extent, to those detected in natural infection, and that infected animals exhibit significantly higher rates of renal dysfunction and histological changes in FIV-infected compared to age-matched, FIV-seronegative animals. Examinations of 326 sick cats from Australia demonstrated a significant association between FIV infection and azotemia and palpably small kidneys [8]. Small kidneys were also reported by Brown and colleagues [9]. Nonspecific renal abnormalities have also been found in other studies [10,11]. Renal alterations in FIV infected cats were observed 5.5% in cats from New Zealand [12], 9.3% in Japan (from a survey of 700 cats) [13], and 9% in 76 cats from three Italian regions (Piedmont, Liguria and Val d’Aosta) [14]. 

In experimentally FIV-infected cats, which were specific pathogen-free, maintained in isolation units, and regularly checked for various clinical and pathological conditions as well as various pathogens, the main alterations observed were mesangial widening with or without segmental glomerulosclerosis and immune-mediated GNs. These renal changes were also observed in naturally FIV-infected subjects though renal amyloidosis and the presence of interstitial infiltrates seemed to occur only in this latter group. Immune-mediated GNs were observed in 12/51 experimentally and in 3/21 naturally FIV-infected cats. Although the incidence of these immune-mediated alterations seems higher in doubly infected animals, the numbers are too small to draw any certain conclusion. The incidence however does not appear to be related to the infecting strain.

Although FIV-infected cats often present hypergammaglobulinemia, which is believed to be triggered by chronic polyclonal B-cell activation [15] and consequent production of immune complexes [15,16], immune-mediated GNs are no frequently reported in FIV infection. In a previous study on 15 naturally FIV-infected cats only one subject showed IgG deposits in mesangial areas [6]. 

Mesangial widening with or without segmental glomerulosclerosis was also detected in experimentally and naturally FIV-infected cats. These alterations [6] as well as nephrosclerosis [11] and thickened Bowman’s membrane [9] have been already reported in natural FIV infections. Such damage is caused by glomerular reactions, which have also been observed in many other, apparently unrelated, clinical entities. These alterations are thus thought to result from intraglomerular hemodynamic alterations [17]. Hemodynamic alterations in FIV infection might be triggered by a sustained production of lymphokines and/or other host and/or viral factors that stimulate mesangial proliferation, and alter glomerular capillary permeability. Although controversial, there is increasing evidence for a direct viral role on renal cells either as the result of exposure to viral proteins or direct infection renal parenchyma [7]. On the other hand, tubular and interstitial lesions due to lymphocytes and plasma cell interstitial infiltration, fibrosis and tubular degenerative changes have been mainly detected in naturally FIV-infected cats [6,9,11,18] and only occasionally in experimentally infected animals. Our study also confirmed previous observations showing glomerular and interstitial amyloidosis in the kidneys of naturally infected animals [7,18,19]. In contrast, none of the 51 experimentally infected cats examined had amyloid deposits in the kidneys. Histochemical and immunohistochemical studies demonstrated that the amyloid deposits were related to secondary amyloidosis, which is typically associated with chronic infections. As a further contribution to the pathogenetic mechanisms of renal disease under FIV infection and seeing that the experimentally FIV-infected cats had no amyloid deposition, the present study would seem to demonstrate that FIV infection alone is not sufficient.

Clinical and pathological studies evidenced that mild to severe renal proteinuria without clinical signs of azotemia is more likely during FIV infection [6]. In a recent study, performed among client-owned cats, no association was detected between FIV infection and renal azotemia was found but 25% (16/64) of naturally FIV-infected cats were proteinuris compared to FIV-uninfected cats (10.3%, 20/195) [20]. Also in our study, only 1/51 experimentally infected animal by FIV-M2, sacrificed one year pi, showed azotemia (serum creatinine 144 µmol/L and mild proteinuria 3.9 g/L with urine protein concentration (UPC) 0.43) (Chronic Renal Failure stage 2) [21]. Renal proteinuria, diagnosed by UPC, was present in 10/17 (56.5%), 9/28 (32.1%), and 2/6 (33.3%) cats infected with FIV-Pet, FIV-M2, and FIV-Pet + FIV-M2, respectively and regardless of the time from infection. However, the most severe renal proteinuria was observed in the FIV-M2 group (mean 3.27 ± 6.34; ranged 0.53–17.63), it was milder in the FIV-Pet + FIV-M2 (mean 3.0 ± 4.39; ranged 0.55–13.01), and was the least severe in the FIV-Pet group (mean 2.56 ± 3.03; ranged 0.31–7.02). A total of 14/29 cats with no histological renal alterations had the lowest mean uP value (3.95 ± 1.37 g/L (2.5–7.05)) and the lowest mean UPC value (0.45 ± 0.12 (0.25–0.67)). Mean uP and UPC values in 10 proteinuric cats with mesangial widening were slightly higher 3.54 ± 1.52 g/L (2.0–5.4) and 0.81 ± 0.44 (0.53–1.94), respectively. Five cats with similar mesangial alteration were aproteinuric (>2.0 g/L). Significantly higher mean uP (21.53 ± 29.39 g/L (2.8–100.0)) and UPC values (4.8 ± 5.77 (0.90–17.63)) were found in 12 cats with glomerular alterations. All 20 cats with tubular alterations had either GN (9/20) or mensangial widening (11/20). Three of them were aproteinuric. Finally, 4/6 cats with interstitial infiltrates were proteinuric, two were infected by 24 months and two by ≥36 months. Electrophoresis of urine proteins confirmed the correlation between proteins excreted in the urine and the histological alterations found in the observed cats: 3/21 (14.3%) cats had glomerulo selective proteinuria, 15/21 (71.5%) had glomerulo non-selective, and 3/21 (14.3%) manifested glomerulo non-selective and tubular proteinuria. 

Most experimentally infected cats (71.4 to 100%) had inverted CD4^+^/CD8^+^ T-cell ratio that depended on the infecting viral isolate and, albeit with low or no statistical significance, time of infection. No correlation between CD4^+^/CD8^+^ T-cell ratio and renal alteration was found. Likewise, CD4^+^/CD8^+^ T-cell ratio and CD4^+^ T-cell counts did not appear to be related with azotemia and proteinuria. This seems to be in agreement with what has been observed in HIV positive patients [9].

Our findings indicate that FIV infection can lead to nephropathy. At glomerular level mesangial cell hyperplasia, focal/segmental glomeruloscerosis, global glomerulosclerosis, reactive visceral epithelium, “collapse” capillaries and dilated Bowman’s space have been described as being associated with FIV and HIV infections as well as foot process effacement, wrinkling of glomerular basement membrane, endothelial tubulareticular inclusions, tubular and interstitial nuclear bodies and chromatin degeneration by electron microscopy [6,22]. This would suggest that FIV and its natural host could be a useful animal model to investigate lentiviral-induced renal diseases.

Autopsy and biopsy series demonstrated that GNs are present in 10–80% HIV infected patients with renal disease [22,23,24,25,26]. These GNs take manifest in various histologic forms including proliferative, lupus-like, and mixed proliferative or sclerotic forms [23]. Other types of GNs, including the membranoproliferative, have also been reported in HIV infection [23]. These renal alterations, however, have not been firmly linked to HIV infection: in fact, they may be consequence of coexisting infections [27,28,29], deranged immune response response to coexisting infections [30], or simply coincidence [24]. It has been demonstrated that the immune response to HIV infection could culminate in specific HIV-immune-mediated GN [31]. Our study, which was carried out with cats experimentally infected by FIV and kept in isolation units confirmed that similar immune-mediate alterations can take place and complicate lentiviral infections.

Another renal disorder believed to be associated with HIV infection is HIV-associated nephropathy (HIVAN). HIVAN patients may develop a spectrum of renal pathologies that are typically manifested with an acute and rapid loss of renal function, along with a presence of proteinuria, nephrotic syndrome, and azotemia [32]. The pathognomonic pathologic findings are characterized by focal segmental glomerulosclerosis, including collapsing glomerulopathy [33,34]. 

Although the pathogenetic mechanisms of HIVAN are mostly unknown, there is evidence to support a causative role of the lentiviral infection. In fact, HIVAN can be reproduced in HIV-1 positive transgenic mice and rats, and non-human primates [35,36,37,38]. In fact, under highly active antiretroviral therapy some patients experienced reversal renal histologic and laboratory abnormalities as demonstrated in small clinical studies of patients with biopsy-proven HIVAN [39,40]. Interestingly, glomerulosclerosis, tubulointerstitial disease and/or mesangial widening with IgM and C3 and mild IgG deposition have also been observed in naturally FIV-infected cats [6] and, as demonstrated by the present study, also in experimentally conditions though in a smaller proportion of infected subjects than in naturally infected cats.

Studies carried out on renal tissues of HIV-infected patients have demonstrated the presence of HIV DNA [23]. The recent identification of HIV-1 messenger RNAs in renal tissues and particularly in glomerular and tubular epithelial cells supported the idea that HIV can replicate in this body compartment [41,42,43]. The hypothesis that FIV may play a role in the pathogenesis of renal alterations is supported by the presence of p24 viral antigen in tubular epithelial cells, by the detection of FIV *gag *DNA and RNA sequences in nucleic acids extracted by kidney biopsies, and by the presence of scattered interstitial inflammatory and glomerular cells [7,14,31]. Studies in transgenic models suggest that HIV-1 regulatory proteins Vpr and Nef play an important role in HIVAN etiopathogenesis [2]. Since FIV has no known functional Vpr and Nef homologs the mechanism underlying renal alterations in FIV infection could be different. A possible explanation is that FIV Vif and/or ORF-A genes may replace some functions of HIV-1 Vpr [2,44]. Recent studies of HIV have shown that deletions of both Vpr and Vif are required to completely prevent G2 “stalling”, a process in between complete G2 arrest and normal cell-cycle progression [45,46]. Similar results were also observed by deleting FIV ORF-A [44]. These results suggest that FIV Vif and ORF-A may overlap some HIV Vpr and Nef functions and thus contribute, with an unknown mechanism, to the induction of renal alterations.

## 3. Experimental Section

### 3.1. Studied Subjects and Experimental Design

Fifty-one specific pathogen-free queens, infected with FIV-Pet and/or FIV-M2 isolates at one year of age and four age-matched controls, were included in the study; 17 cats were inoculated with FIV-Pet isolate alone (group FIV-Pet), 28 with FIV-M2 isolate alone (group FIV-M2), and six with FIV-Pet and FIV-M2 (group FIV-Pet + FIV-M2). All subjects were aged between 2 and 6 years at the time of analysis (median age of 8 years, range 2–6 years). Infected and control cats were housed in biosafety hazard level 3 conditions at the Retrovirus Center of the University of Pisa, and were monitored daily for clinical conditions throughout the observation period. Physical examination was performed weekly for the first two months pi and then monthly. FIV-Pet isolate was obtained from the supernatant of persistently infected FL4 cells [47] but prepared differently for the two groups. Cats from the FIV-Pet group infected with a virus obtained by serial passages in SPF cats to increase viral pathogenicity. Animals were inoculated intravenously at 10 CID_50_. The FIV-Pet used to infect the group FIV-Pet + FIV-M2 group derived from FL-4 supernatant and was inoculated intraperitoneally in scaled doses, as described in [5]. FIV-M2 is a local isolate used for vaccine studies and is not cultured *in vitro* [21]. Cats from the FIV-M2 and FIV-Pet + FIV-M2 groups were inoculated intravenously with FIV-M2 doses ranging from 10 to 30 CID_50_. Of the 17 animals in the FIV-Pet group, six were sacrificed at 12 months pi, seven at 24, and four at ≥36 months pi. The 28 animals of FIV-M2 group were sacrificed at 12 months pi (10 subjects), 24 months pi (6 subjects) and ≥36 months pi (12 subjects). Finally, the six animals in the FIV-Pet + FIV-M2 group were first inoculated with the *in vitro*-grown FIV-Pet and, one year later, superinfected with FIV-M2. Animals were sacrificed two years after FIV-M2 superinfection. All cats were routinely examined for CD4^+^ and CD8^+^ T lymphocyte subsets by flow cytometry, various chemical and physical parameters, immunological responses and viremia and proviral load as described in [5,48]. All animals seroconverted in 4–6 weeks. Four uninfected SPF cats were used as negative controls (group C). Histology was performed at the indicated times pi in all animals that were heavily anesthetized and then euthanized for necropsy.

The naturally FIV-infected cats came from different part of Tuscany, Italy. The median age of these subjects was 8 years (range 4–13 years). Even if it was not possible to precisely determine the duration of infection in these animals, the eleven symptomatic cats were most likely infected by over 36 months. FIV infectious status was established by detecting antibodies to FIV by a commercial enzyme linked immunosorbent assay (ELISA; Idexx, Portland, ME, USA), confirmed by western blot analysis and by PCR analysis in PBMC as previously described [5,15]. All FIV-infected subjects selected were negative for feline leukemia virus p27 antigen by a commercial ELISA (Idexx), and feline infectious peritonitis antibodies (Diasystems Celisa FIP, Tech America, Omaha, NE, USA). The eleven animals shown full-blown F-AIDS were sacrificed with the owner consensus. The ten cats in the asymptomatic phase died for unrelated causes and were submitted to necropsy. At the time of sacrifice, all animals had a marked reduction of CD4^+^ T-cell levels and an inversion of CD4^+^/CD8^+^ T-cell ratio.

### 3.2. Biochemistry and Urine Analysis

Urine specimens were obtained by cystocentesis. After centrifugation, supernatants were used to determine protein and creatinine concentrations using two commercial assays (BioRad, Richmond, CA, USA, and the Creatinine-Jaffe method, Verbena, Milano, Italy, respectively). In cats with marked proteinuria (>2 g/L), UPC was calculated using the following formula: P(g/L) × 100/(Cr mmol/L/0.0885) Protein qualitative analysis was performed with sodium dodecyl sulfate-polyacrylamide gel (SDS-PAGE) according to Laemlli [49]. Proteins were divided in low molecular weight proteins (LMW < 66 kDa) and high molecular weight (HMW > 76 kDa). LMW proteins were considered tubular, HMW together with increased UPC value >0.4 were considered glomerular proteinuria.

Blood samples for biochemical profile were collected into serum separator tubes (Vacuette, Greiner Bio-One, Kremsmunster, Austria) and left for 30 min at 4 °C to clot. Samples were then centrifuged (1,300 g for 10 min) to separate and collect the serum. Serum samples were assayed for various biochemical parameters including urea, creatinine, total protein, and albumin. Analyses were performed with a spectrophotometer (LKB Biochrom Ltd., Cambridge, UK). 

### 3.3. Molecular Analysis

Buffy coat from 2 mL whole blood samples was used to detect and quantitate FIV provirus. FIV DNA extraction and amplification was performed by real-time PCR [48] for group 1 and 2 cats, and competitive PCR [50] for group 3 cats. Both assays are designed on FIV gag sequences and when tested in parallel to update technology from older competitive to newer real-time PCR assay, gave similar results [48]. All precautions were taken to avoid contamination and samples were examined at least twice in separate experiments. DNA from the PBMC of uninfected cats and FIV-Pet infected FL-4 cells were used as negative and positive controls, respectively.

### 3.4. Flow Cytometry

T-cell subsets were examined by flow cytometric analysis as described [51]. Flow cytometry analysis was performed using fluorescein conjugated murine monoclonal antibodies to feline CD4 and CD8 T-cells surface markers (Southern Biotech, Birmingham, AL, USA) and an Epics Elite cell analyzer (Coulter Electronics, Hialeah, FL, USA).

### 3.5. Histological Investigations

Renal tissue samples were fixed in 10% buffered formalin solution and embedded in paraffin. Histopathological examination was performed by AP at the Department of Animal Pathology, Prophylaxis and Food Hygiene, and without knowing the infection status of the examined animal. Three-µm thick sections from each specimen were stained with hematoxylin and eosin, periodic-acidic Schiff, Jones periodic acid-silver methenamine and Azan trichrome. Sections with inflammatory lesions were stained with Ziehl-Neelsen acid-fast and Gram to prevent bacterial infections. Amyloid deposits were detected by alkaline Congo red staining with polarization on 8 µm sections [52]. Differentiation between primary and secondary amyloidosis was based on staining by a modified Romhanyi method with pre-treatment with potassium permanganate [53] and IHC using antibodies against the amyloid A protein.

### 3.6. Immunohistochemical Investigations

The localization of IgG, IgA, IgM and C3 deposits was investigated by indirect IF and IHC. IF was performed using as primary antibodies primary sheep monospecific antibodies to cat IgG, IgM, IgA and C3 (Binding Site, Birmingham, UK) and a rabbit fluorescein anti-sheep IgG (Vector Laboratories, Burlingame, CA, USA), as described [6]. Control sections were incubated with normal sheep serum (Dako, Golstrup, Denmark) before treatment with the secondary antiserum. For IHC, sections were de-waxed in xylene, passed through a graded series of alcohols, and rehydrated in deionised water. For Ig and C3 localization, the tissues were digested with 0.5% protease (Protease XXIV; Sigma, Saint Louis, Mo, USA) in 0.05 M Tris-HCl, pH 7.6. Endogenous peroxidases were exhausted with 0.5% hydrogen peroxide for 30 minutes followed by three washes in 0.05% Tween Tris Buffered Saline solution (TBST) at pH 7.6. Normal serum from the respective secondary antibody host species and diluted 1/10 in TBST was added to the tissue sections and incubated at room temperature for 30 minutes. After three washes, TBST diluted primary antibodies were applied to the sections and incubated at room temperature for one hour. The antisera used in IHC were produced in sheep (polyclonal anti cat IgG, IgA, IgM and C3), mice (monoclonal against the human AA protein and cross-reacting against the homologous cat protein), and rabbit (polyclonal against anti-cat AA and AL; a kind gift from R. P. Link, University of Munich, Munich, Germany). After three washes, the secondary biotinylated antibody (Vectastain, Vector Labs Inc., Burlingame, CA, USA) was added and incubated at room temperature for 30 minutes. Peroxidase reaction was developed for 10 minutes using diaminobenzidine (Impact DAB, Vector Labs Inc., Burlingame, CA, USA) and blocked with deionised water. Negative controls were performed by replacing the primary antibody with normal sheep or rabbit serum, or using an irrelevant murine subclass matched (IgG_1_) monoclonal antibody. 

### 3.7. Statistics

Statistical analysis was performed using the statistical package SPSS Advanced Statistics 13.0 (SPSS Inc., Chicago, IL, USA). Serum creatinine concentrations, UPC and lymphocyte subset counts were not normally distributed, therefore comparison of median values was performed using the Mann-Whitney test. A Chi-square test was used to investigate the significance of the relationship between protein expression and individual variables. Statistical significance was based on a 5% (0.05) significance level. 

## 4. Conclusions

Our study confirms that renal involvement occurs in a high proportion of naturally FIV-infected cats and that these alterations are, although to a lesser extent, also found in experimentally infected subjects. Since the latter animals were maintained in isolated units and were therefore shielded from external factors known to exacerbate FIV infection, these results suggest a causative relationship between FIV infection and renal abnormalities. This damage seems to be related to a mesangial increase, sometimes accompanied by mesangial cell proliferation and glomerulosclerosis, a lower percentage of immune mediate GN and, in naturally infected subjects, glomerular and interstitial amyloidosis. These alterations are fir the most part similar to those detected in HIV-infected patients. FIV and its natural host, the domestic cat, is therefore an interesting natural animal model to study the pathogenesis of HIVAN and renal alterations associated with chronic lentiviral infections.
